# Advertisers, &c.

**Published:** 1889-02

**Authors:** 


DEMONOLOGY OR SPIRITUALISM, Ancient and Modern, by Eld. E. F. Hanson of the Maine Eldership of the church of God—Belfast, Maine—J. E. Jewett Pub. 77 Bible House, N. Y.

This is a work of 310 octavo pages in which the dark side of Modern Spiritualism is fully and forcibly set forth. The phenomena are not denied, but the evils and dangers of the system are disclosed with no sparing hand. The dedication of the book is in these words: "To the readers of this book, who may desire to know the truth and nothing but the truth concerning the subject herein disclosed, the following pages are affectionately inscribed":

The phenomena of Modern Spiritualism are attributed to Demus, and it is admitted that they can and do communicate with mortals—there is no possible guarantee and no certainty as to the identity of the spirit. No way or possibility of being assured as to who they are. There is no definite theory or religious belief; having the power of reading the mind they can ascertain the history and facts in the life of every one who subjects himself to their influence and can assume ihe names and personate with amazing accuracy the peculiarities of one departed friend and thereby deceive almost any one who places himself in rapport with them.

The book shows that the phenomna are not new but have existed in all countries from remote times, and are unquestionably the work of the devil, and should therefore be avoided by all men. Many facts are given also in regard to the witchcraft as it prevailed in the early settlement of the American Colonies, and much interesting information concerning the history of these strange phenomena is given.

THE VEST POCKET ANATOMIST. (Founded upon "Gray.") By C. Henri Leo nard, A. M., M. D., Professor of the Medical and Surgical Diseases of Women and Clinical Gynæcology in the Deiroit College of Medicine. *Fourteenth revised edition*, containing 193 illustrations, "Dissection Hints" and Visceral Anatomy. Cloth, 12mo., 304 pages; price $1.00. ILLUSTRATED MEDICAL JOURNAL Co., Publishers, Detroit, Mich.

The new fourteenth edition of this work has been increased in size by the addition of over 100 pages of text and one hundred engravings; the page of the book has also been somewhat enlarged to accommodate better the engravings. The brain and its Membranes, the Eye, Ear and Throat, in fact the entire Viscera and the Generative organs of both sexes, form the new subject matter in this edition. Besides being a very popular dissecting room companion, it has become also a very popular surgical case companion for the practitioner, since the illustrations show at a glance (being photo-engraved from the English cuts of Gray) the positions of all the important blood vessels, nerves, muscles and viscera.

J. B. DANIEL, DRUGGIST.—Be sure and see the new and interesting advertisement of Jno. B. Daniel of Atlanta. Mr. Daniel is an energetic, accomodating and reliable gentleman. Call and see him opposite entrance to Car Shed.

SPECTACLES.—A good place to get spectacles is at the establishment of Er Lawshe at 47½ Whitehall street. Mr. Lawshe may well be termed the "Old reliable," being one of the oldest and most reliable business men in the City of Atlanta. You will find him up stairs, and what he tells you about spectacles you may depend upon.

NEEDLESS FEARS.—In an able and interesting address delivered before the Penn. Med. Society, June, 1888, by T. Green Easton Pa., [Sanitarian] we find a number of interesting points in regard to what may be termed needless fears. Among these, several cases are cited to show that even death itself, so far as physical suffering is concerned, ought not to excite the grave naxieties which so many feel and which haunt the lives of timid and nervous people. Shakespear, in the play of Julius Cæsar makes one of the characters say "He that cuts off 20 years of life cuts off so many years of fearing death."

In the epistle to the Hebrews Chap. 2 : 15, in referring to the fears that chrst came to allay we find these words : "And deliver them who through fear of death were all their life time subject to bondage."

As it relates to physical suffering, the manner of death by the brain, the lungs or the heart, is sufficient to prove that there is unnecessary fear of suffering in the last hour.

The Scripture account of death usually is; "He fell asleep." The Greek word, koimeterion, from koimao, "I sleep," from which our word cemetery is derived, beautifully expresses the Scripture idea, a sleeping place.

There are no cases of death in which the suffering is so certain as in pseudo-membranous laryngitis. Dr. J. C. Rushmore, of Brooklyn, in the New York *Medical Journal*, May 9th, 1888, p. 542, writes: "In regard to the history of the case, if the operation (tracheotomy) is not done, my experience has been that death is not nearly so painful as I was at first led to suppose. I make this statement with some hesitation, in view of the fact that many authors advise the operation on the ground that even if the case has a fatal termination, the distress is less than without it. I am speaking now, of course, of those cases where dyspnœa is due to mechanical causes. When patients have died from laryngeal obstrucion alone, the picture has always been the same—gradually increasing restlessness and dyspnœa, with paroxysms added at times, and threatening death; then the spasm is relieved in a few moments, but a very considerable amount of distress continues, and then a rather rapid development of unconciousness, the coma continuing for several hours, and the patient dying quietly, the breathing being still obstructed. And this is so uniform in my experience, that I have been in the habit of telling the patients' friends that, even if the operation is not done, the patient will not choke to death, with great struggling and distress, but will die unconscious and with comparative ease.

Hon. James T. Nisbet has bean elected a trustes of the Southern Medical College in place of John Keely diseased.

Ex Governor Boynton, of Griffin, Ga., was chosen to fill the vacancy occasioned by the death of the Hon. D. W. Lewis.

SOUTHERN MEDICAL COLLEGE COMMENCEMENT.—The commencement exercises of the Southern Medical College is appointed for the evening of the 2nd of March next at the Opera House. It is expected that the occasion will be an unusually attractive one. The Profession and citizens generally are invited to be present.

